# Immune cell profile and metabolic preference following intramuscular lipopolysaccharide injection of highly inbred and advanced intercross genetic lines

**DOI:** 10.3389/fvets.2025.1592021

**Published:** 2025-06-03

**Authors:** Kayla M. Elmore, Susan J. Lamont, Elizabeth A. Bobeck

**Affiliations:** Department of Animal Science, Iowa State University, Ames, IA, United States

**Keywords:** cellular metabolic profile, C-reactive protein, genetic line, immune cell profile, immunometabolic assay, lipopolysaccharide, poultry

## Abstract

Lipopolysaccharide (LPS), a gram-negative bacterial cell wall component commonly used in animal models of inflammation, is also universally found in poultry environments. Documented LPS effects in production animals include reduced feed intake and weight loss; however, research into LPS’s impact on cellular metabolism and immune recovery is limited. This study compared baseline and stressed metabolic phenotypes of peripheral blood mononuclear cells (PBMC) from highly inbred genetic lines and examined fuel preference, cell profiles, and C-reactive protein (CRP) expression at baseline and post-LPS injection. Forty birds from 4 genetic lines (Ghs, Line-8, Sp-21.1, and AIL-F) were randomly assigned to 1 of 2 treatments, receiving intramuscular injections of saline or 1 mg/kg BW LPS (*Escherichia coli* O55: B5). Body weight was recorded before injection (baseline) and 24 h post-injection (hpi), with cloacal temperature recorded at baseline, 6 hpi, and 24 hpi. Blood was collected at all timepoints for PBMC isolation, metabolic analysis, flow cytometry, and plasma CRP ELISA. Statistical analysis used the SAS 9.4 MIXED procedure with fixed effects of genetic line, injection status, and their interaction followed by Tukey–Kramer adjustment, with significance denoted at *p* ≤ 0.05. Baseline immune profiles and ATP production varied by line (*p ≤* 0.02). LPS did not significantly impact body weight or temperature but influenced all immune cell populations and CRP concentration at 6 hpi (*p ≤* 0.02). Sp-21.1 exhibited a glycolytic metabolic profile and higher baseline CD3^+^CD1.1^+^ and CD3^+^CD4^+^ populations, suggesting enhanced antigen presentation and cytokine signaling. AIL-F displayed sustained monocyte/macrophage activation post-LPS and the highest baseline CD3^+^CD8α^+^ populations, indicating a distinct cytotoxic immune response. Line-8 maintained the highest CD3^+^ populations post-LPS and increased ATP production at 6 hpi, suggesting a balance between immune activation and metabolic compensation. Ghs exhibited a depletion of monocyte/macrophage^+^ cells post-LPS but later recovered, highlighting a delayed immune response that may impact pathogen resistance. Results suggest genetic line may have a greater influence on metabolic pathway preferences than LPS injection in this experiment. Characterizing metabolic changes during immune activation and recovery may offer insights into breed-specific production traits and inform future breeding and management strategies to enhance health and production efficiency.

## 1 Introduction

Lipopolysaccharide (LPS) is a major structural component in the outer membrane of gram-negative bacteria, such as *Escherichia coli*, which can be highly immunogenic upon host infection (1). In livestock systems, LPS endotoxins can be ingested through contaminated feed and water sources or inhaled from aerosolized dust and feces. Once in systemic circulation, toll-like receptor 4 (TLR4) recognizes LPS through LPS binding protein and cluster of differentiation 14, which deliver LPS to myeloid differential protein 2 (MD2) on the cell surface (2). TLR4 then dimerizes with MD2, initiating a signaling cascade through two distinct pathways: myeloid differentiation factor 88 (MyD88) -dependent pathway and MyD88-independent pathway (2–4). The MyD88-dependent pathway leads to the activation of nuclear factor-kappa B and mitogen-activated protein kinases, which drives pro-inflammatory cytokine production, such as interferon-gamma (IFNγ), tumor necrosis factor *α* (TNFα), and interleukin-6 (IL-6), and promotes heterophil and macrophage activation (2, 3). The MyD88-independent pathway is activated through TIR-domain-containing adapter-inducing interferon-*β* and leads to dendritic cell activation and IFNβ promotion (2, 4). These pro-inflammatory cytokines stimulate the release of C-reactive protein (CRP) into the bloodstream, making CRP a useful marker for assessing systemic inflammation (5).

Animal health and performance are dependent on the innate immune system to serve as the first line of defense against pathogens and infectious diseases. LPS can stimulate both acute and chronic inflammatory pathways. Prolonged immune system stimulation can lead to physiological and metabolic stress due to increased energetic demands (6, 7). In poultry, downstream physiological responses to LPS infection include fever response and decreased activity, feed consumption, weight gain, and egg production (8, 9). Therefore, LPS is a widely used sterile inoculant model to study systemic inflammation. In avian models, a dosage of 1 mg/kg BW LPS injected intraperitoneally has been shown to induce inflammation as measured in various contexts, including fever, intestinal inflammation, and immune outcomes (10, 11). Meanwhile, broilers may need an increased 5 mg/kg dose compared to 1 mg/kg layer-type birds to elicit a similar immune response (11). Other researchers have defined broilers as relatively LPS resistant, with lethal intravenous LPS dosage reaching 50 mg/kg BW (12). Alternative routes of LPS administration have also been shown to impact physiological and immune outcomes. For example, an intramuscular LPS dose of 2 mg/kg has also been shown to elicit weight loss and fever response in cockerels and pullets within 24 h of administration (13). Additionally, previous *in vitro* studies have shown enhanced energy metabolism via glycolysis following an LPS injection (14, 15). However, research investigating comparative changes in cellular metabolism under stressed conditions, such as LPS injection, within an *in vivo* avian model is lacking.

Genetics plays a significant role in immune cell functionality and disease resistance. In addition to investigating the impact of LPS injection on immunometabolic processes and downstream physiological outcomes, comparing genetic lines with varying tolerance to LPS may provide a better understanding of how cellular metabolic outcomes shift following systemic challenge. Previous studies using Newcastle Disease virus (NDV) and Avian Influenza virus (AIV) models have demonstrated that highly inbred chicken lines, such as the Fayoumi line, exhibit stronger immune defenses against specific diseases (16–19). For instance, two Fayoumi lines (M-5.1 and M-15.2) have been shown to have higher resistance to LPS compared to the Leghorn Ghs-6 line (20, 21). Overall, the Fayoumi lines are known for their higher resistance to bacterial and viral infections than the heritage Leghorn lines (17–24). While existing research has focused on the Fayoumi and Leghorn lines, less information is known regarding the immune and metabolic responses of other genetic lines.

The Ghs Leghorn lines originated from two US commercial layer lines and are major histocompatibility complex congenic (18, 25). Ghs-6 is known to be more resistant to AIV than Ghs-13; however, the comparison between the lines using a bacterial infection model has not been previously researched (16). Similarly, Line-8 is a Leghorn line originating from Iowa State University inbred lines and has been shown to have a higher Major Histocompatibility Complex (MHC) class 1 expression which may suggest a more specialized immune response and more adept at specific pathogen recognition (26). Line-8 has also been characterized with increased mitochondrial respiration (OCR) in comparison to commercial broiler and layer lines, but not Spanish-21.1 [Sp-21.1; (25, 27)]. Sp-21.1 originated from Spain and has been inbred since 1954 (25). While its immune-related responses to bacterial infections are relatively unknown, Sp-21.1 has been characterized by low MHC class 1 protein expression, which suggests that it may be similar to the standard B21 MHC type and be more tolerant or less specific in pathogen recognition (26, 28). Similar to the Fayoumi relative, the advanced intercross line (AIL-F), a genetic cross between broiler and Fayoumi lines, is known to be heat resistant, but its metabolic and immune responses following bacterial infection are also not well understood (25, 29).

Therefore, the study objectives were to compare baseline and stressed metabolic phenotypes and cell profiles of isolated PBMC from highly inbred (Ghs, Line-8, and Sp-21.1) and advanced intercross (AIL-F) genetic lines. These lines were included in the present study as they were expected to exhibit varying immune and metabolic responses to LPS stimulation. Additionally, the study aimed to investigate the fuel preference of cells under stressed conditions following an *in vivo* immune system inoculation and *in vitro* assay challenge with metabolic pathway inhibitors and to evaluate plasma C-reactive protein concentrations following LPS injection.

## 2 Materials and methods

### 2.1 Animals and treatments

All experimental procedures were approved by the Iowa State University Institutional Animal Care and Use Committee #22–113. A total of 40 mature (~64 weeks of age) birds from 4 highly inbred (Ghs-6, Ghs-13, Line-8, and Sp-21.1) and advanced intercross genetic lines (AIL-F) were used from a breeding colony maintained at Iowa State University’s Robert T. Hamilton Poultry Teaching and Research Facility (Ames, IA). The lines were selected to represent wide biodiversity within the species and a variety of known response types to avian pathogens and expression levels of MHC antigens (Table 1). Three roosters and 7 hens per genetic line were used from Line-8, Sp-21.1, and AIL-F. Six Ghs-6 and four Ghs-13 birds were included and pooled as the “Ghs” line as they are MHC-congenic lines and were found to not be statistically different across any measures. Birds were housed in individual hanging cages with *ad libitum* access to a standard laying hen diet and water. On d0, 10 birds/genetic line were randomly assigned to one of two injection treatments, resulting in a 4 × 2 factorial of genetic line (Ghs, Line-8, Sp-21.1, and AIL-F) by injection. Each bird received an intramuscular injection evenly divided across the right and left breast and thigh muscles with 1 mg/kg BW lipopolysaccharide (LPS, *Escherichia coli* O55: B5; Sigma-Aldrich, St. Louis, MO) or an equivalent amount of 0.9% sterile saline. Body weight was recorded at baseline immediately before injection and 24 h post-injection (hpi), with cloacal temperature recorded at all time points (baseline, 6 hpi, and 24 hpi).

**Table 1:** Genetic line, immune traits, and description of all genetic lines included in the experiment.

Genetic line	Immune traits	Description
Ghs	Moderate resistance to coccidiosis (42) Less resistant to AIV^a^, NDV^b^, and MDV^c^ than Fayoumi line (17, 18, 24)	Inbred since 1954. Leghorn lines originated from two US commercial layer lines (25, 26).
Line-8	Needs further investigation	Inbred since 1925. Leghorn line originated from crosses of ISU inbred lines (25, 26).
Sp-21.1	Needs further investigation	Inbred since 1954. Spanish line originated from Spain (25, 26).
AIL-F	Heat tolerance (29)	Maintained since the early 2000s. F32 generation from 1 male sourced from a commercial broiler breeder line crossed with 6 Fayoumi females (25).

a AIV = Avian Influenza virus.

b NDV = Newcastle Disease virus.

c MDV = Marek’s Disease virus.

### 2.2 PBMC isolation

Approximately 1 mL of blood was collected from the brachial vein of all birds at all timepoint using heparin-coated syringes and heparinized collection tubes. Following previously published methods (30, 31), PBMC were isolated from 1:1 sterile phosphate-buffered saline (PBS) to whole blood using Histopaque 1,077 and 1,119 density gradient (Sigma Aldrich, St. Louis, MO) and centrifugation. Following two sterile PBS washes and resuspension in Seahorse XF DMEM medium (pH 7.4, 37°C; Agilent, Santa Clara, CA), isolated cells were counted by hemocytometer for immunometabolic assays. Following, the remaining PBMC were frozen in Seahorse XF DMEM medium, 42.5% heat-inactivated chicken serum (Equitech-Bio Inc., Kerrville, TX), and 7.5% DMSO (Thermo Fisher Scientific, Waltham, MA) at −80°C for immune cell profile detection using flow cytometric analysis. In addition, 1:1 diluted plasma to PBS was also collected and frozen at −80°C for use in C-reactive protein enzyme-linked immunosorbent assays (ELISA).

### 2.3 Flow cytometry

Following similar previously published methods (30, 31), frozen PBMC previously isolated at baseline, 6 hpi, and 24 hpi timepoints were thawed, washed in RPMI-1640 medium (Cytiva-Hyclone, Logan, UT), resuspended in PBS (Corning, Corning, NY), and aliquoted evenly across 6 flow cytometry tubes (Corning, Corning, NY) per sample. The flow cytometry panel included the following antibodies to detect innate immune cell and T lymphocyte populations: PE anti-chicken monocyte/macrophage (clone KUL01; mouse IgG1κ), Pacific Blue™ anti-chicken CD3 (clone CT-3; mouse IgG1κ), FITC anti-chicken CD1.1 (clone CB3; mouse IgG1κ), PE/CY7 anti-chicken CD4 (clone CT-4; mouse IgG1κ), Alexa Fluor^®^ 700 anti-chicken CD8α (clone CT-8; mouse IgG1κ; Southern Biotech, Birmingham, AL). All samples were primarily stained with fluorescence-minus-one controls and corresponding isotype controls (0.3/50 μL PBS) for 30 min at 4°C in the dark. Cells were washed, centrifuged, and resuspended in PBS for analysis on a BD FACSCanto™ cytometer (BD Biosciences, San Jose, CA). Cell populations were analyzed using FlowJo version 10.5.0 software (BD Biosciences, San Jose, CA), where singlet-live cell populations were used to identify monocyte/macrophage^+^ and CD3^+^ cells, and CD3^+^ populations were further gated to identify CD1.1^+^, CD4^+^, and CD8α^+^ cells.

### 2.4 Immunometabolic assays

Freshly processed PBMC were plated in triplicate at 200,000 cells/well in a 96-well XF cell culture plate for use in the Seahorse XF Real-Time ATP Rate Assay and Glycolytic Rate Assay kits on the Seahorse XFe96 Analyzer (Agilent, Santa Clara, CA). Assay kit protocols outlined by Agilent User Guides were followed to prepare and perform metabolic tests and the machine temperature was set at 40°C. Briefly, the Real-Time ATP Rate Assay measures the real-time rate of total adenosine triphosphate (ATP) production and pathway-specific ATP production, mitochondrial oxidative phosphorylation (OXPHOS) and glycolysis, using two serially injected metabolic modulators: oligomycin (15 μM) which targets the ATP synthase complex V, inhibiting mitochondrial ATP synthesis (i.e., decreases mitochondrial respiration), followed by a 1:1 mix of rotenone and antimycin A (5 μM, Rot/AA) which targets mitochondrial electron transport chain complexes 1 and 3, respectively, leading to the complete inhibition of mitochondrial respiration (i.e., increases glycolysis). The Glycolytic Rate Assay measures real-time glycolysis rates, including compensatory and residual glycolysis using serially injected 1:1 mix of Rot/AA (5 μM), followed by 2-deoxy-D-glucose (500 mM, 2-DG), which inhibits glucose hexokinase, resulting in glycolysis inhibition. Throughout both assays, Agilent Wave software (version 2.6.1) measures fluctuations in media pH, hydrogen production, and oxygen consumption, allowing results to be computed into ATP production rates (pmol/min) and proton efflux rate (PER; pmol/min).

### 2.5 Plasma CRP assay

A titration experiment was conducted using 1:1 diluted plasma to PBS from several birds at baseline to determine optimal C-reactive protein (CRP) concentration and detection range using Chicken CRP ELISA kits (MyBioSource, San Diego, CA). Frozen plasma was thawed and diluted 16,000-fold, and CRP concentration was assessed on baseline, 6 hpi, and 24 hpi samples. Plasma samples and standards were plated in duplicate at 100 μL/well on pre-coated plates, and O. D. absorbance at 450 nm was read using a microplate reader (Agilent, Santa Clara, CA). Assay kit protocols were followed to prepare reagents and standards, perform tests, and quantify absorbance data.

### 2.6 Statistical analysis

All data were analyzed using the following model:


$$y_{\mathrm{ijk}}=\mu +G_{i}+C_{j}+{\left(G\;\times \;C\right)}_{\mathrm{ij}}+\in_{\mathrm{ijk}}$$

where y_𝑖𝑗k_ is the observed effect (BW, cloacal temperature, metabolic response, cell population, and CRP concentration) at each timepoint, 𝜇 is the overall mean value, G_i_ is the genetic line main effect for the 𝑖^𝑡ℎ^ level (𝑖 = 4; Ghs, Line-8, Sp-21.1, or AIL-F), C_j_ is the injection main effect for the 𝑗^𝑡ℎ^ level (𝑗= 2, control or LPS), and (G x C)_ij_ is the genetic line and injection interaction. The UNIVARIATE procedure was used to identify and exclude outliers in each data set. All data were analyzed within each timepoint using a mixed linear model with Tukey–Kramer adjustment to account for multiple comparisons (PROC MIXED, SAS 9.4, Cary, NC). Fixed effects of genetic line, injection status, and the genetic line and injection interaction were analyzed for all measures at each timepoint (baseline, 6 hpi, and 24 hpi). The random effect of the tubes was used for flow cytometric data. The least-square means and standard error (SEM) were reported for all measures. Significance was denoted at *p* ≤ 0.05, and trends were reported at a *p*-value between 0.05 and 0.10.

## 3 Results

### 3.1 Body weight and temperature

LPS injection did not significantly affect bird body weight at any timepoint. The LPS injection numerically reduced cloacal temperature by 0.27°C in LPS-stimulated birds compared to control at 6 hpi (*p* = 0.06, Table 2), with no differences at 24 hpi (*p* ≥ 0.05). Body weights among the genetic lines used in the study are known to be different as a characteristic of genetic line and, therefore, are not discussed here. No mortalities occurred during the experiment.

**Table 2:** Body weight and temperature by genetic line ± 1 mg/kg intramuscular LPS injection at all timepoints.

Measure	Ghs		Line-8		Sp-21.1		AIL-F		Adj. *p*-value		
	Control	LPS	Control	LPS	Control	LPS	Control	LPS	Line^1^	Trt^2^	Line^1^ x Trt^2^
BW (kg)											
Baseline	1.62^b^	-	1.48^b^	-	1.55^b^	-	2.80^a^	-	<0.0001	-	-
Pooled SEM	0.13	-	0.13	-	0.13	-	0.13	-	-	-	-
24 hpi	1.50	1.62	1.44	1.47	1.53	1.47	2.60	3.00	<0.0001	0.36	0.64
Pooled SEM	0.19	0.19	0.19	0.19	0.19	0.19	0.19	0.19	-	-	-
Δ24 hpi	0.01	0.08	0.02	0.02	0.02	0.05	−0.01	0.03	-	-	-
SEM	0.15	0.14	0.14	0.20	0.14	0.21	0.38		-	-	-
Temperature (°C)											
Baseline	40.82	-	41.14	-	41.11	-	40.99	-	0.12	-	-
Pooled SEM	0.10	-	0.10	-	0.10	-	0.10	-	-	-	-
6 hpi	41.41	40.70	41.19	40.69	41.09	41.18	40.84	40.90	0.54	0.06	0.11
Pooled SEM	0.19	0.19	0.19	0.19	0.19	0.19	0.19	0.19	-	-	-
24 hpi	41.12	40.88	41.13	40.93	41.08	41.00	40.83	40.84	0.70	0.35	0.91
Pooled SEM	0.19	0.19	0.19	0.19	0.19	0.19	0.19	0.19	-	-	-
Δ6 hpi	−0.59	0.12	−0.04	0.46	0.02	−0.07	0.17	0.07	-	-	-
SEM	0.21	0.36	0.14	0.16	0.18	0.17	0.14	0.09	-	-	-
Δ24 hpi	−0.31	−0.06	0.01	0.21	0.03	0.11	0.16	0.15	-	-	-
SEM	0.27	0.26	0.18	0.15	0.19	0.17	0.16	0.15	-	-	-

Data represent the mean ± SEM (*n* = 10 birds/genetic line at baseline and *n* = 5/treatment of each genetic line at 6 hpi and 24 hpi). Different letter superscripts across rows are significantly different (*p* ≤ 0.05).

1 Line = Genetic line main effect.

2 Trt = Injection main effect.

### 3.2 Immune cell populations

**Figure 1:**
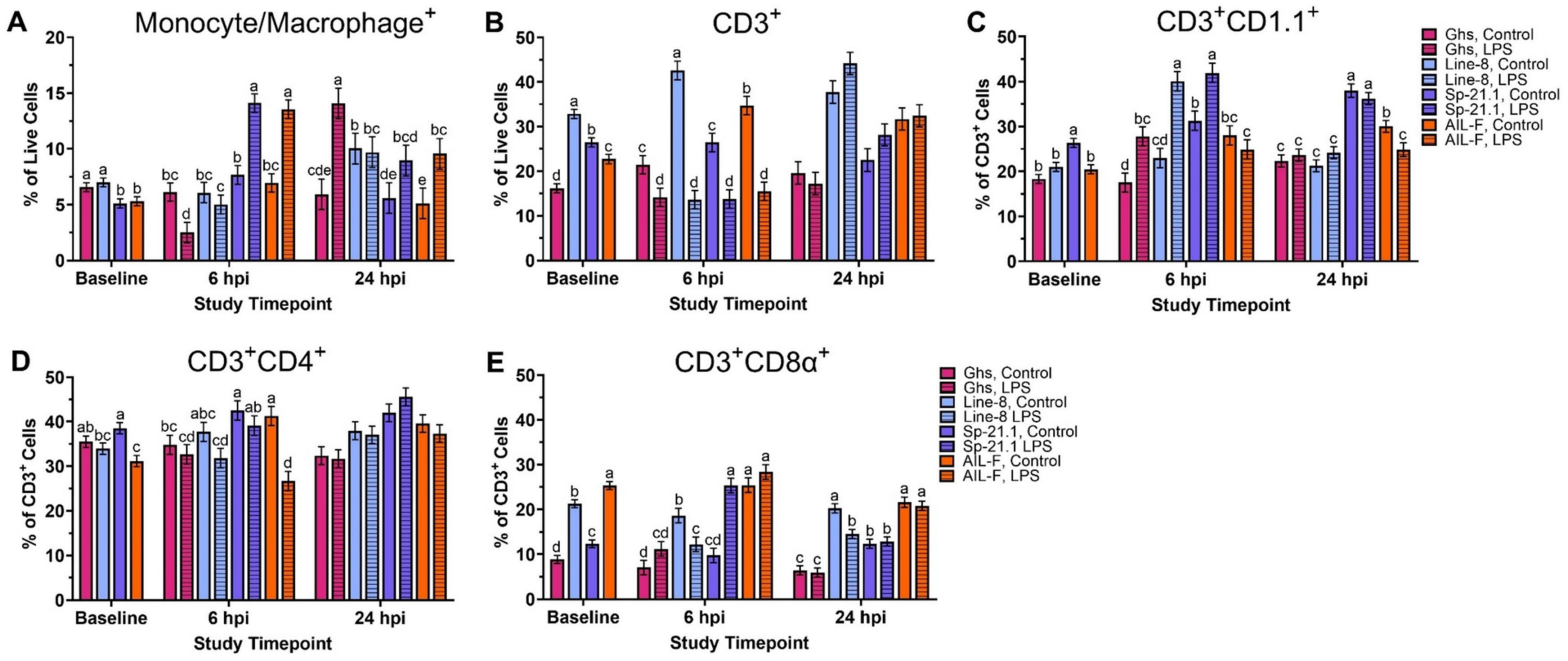
Percentages of **(A)** Monocyte/Macrophage^+^, **(B)** total CD3^+^, **(C)** CD3^+^ CD1.1^+^, **(D)** CD3^+^ CD4^+^, and **(E)** CD3^+^ CD8α^+^ cells isolated from peripheral blood mononuclear cells of genetic line (Ghs, Line-8, Sp-21.1, and AIL-F) ± 1 mg/kg intramuscular LPS injection at baseline, 6 hpi, and 24 hpi. Data represents the mean ± SEM (*n* = 10 birds/genetic line at baseline and *n* = 5/treatment of each genetic line at 6 hpi and 24 hpi). Note that the y-axis is larger in panels **(B-E)**. Different letter superscripts denote significant differences within a timepoint *p* ≤ 0.05.

Baseline PBMC populations were found to be different based on genetic line (*p* ≤ 0.002; Figure 1). Ghs and Line-8 birds had 23.3% more monocytes/macrophages^+^ cells, on average, when compared to Sp-21.1 and AIL-F birds at baseline (*p* = 0.002, Figure 1A, Supplementary Table 1). Line-8 birds also had more CD3^+^ cells at baseline than all other lines (50.9% Ghs, 19.5% Sp-21.1, and 30.9% AIL-F, respectively; *p* < 0.0001; Figure 1B). In CD3^+^ cell subpopulations at baseline, Sp-21.1 had 30.3, 20.2, and 22.2% more CD3^+^CD1.1^+^ cells than Ghs, Line-8, and AIL-F lines, respectively (*p* < 0.0001; Figure 1C). Sp-21.1 birds also had higher levels of CD3^+^CD4^+^ cells by 11.8 and 19.1% compared to Line-8 and AIL-F birds, respectively, while the Ghs line was similar to Sp-21.1 and Line-8 but had 12.2% more CD3^+^CD4^+^ cells than AIL-F (*p* = 0.001, Figure 1D). AIL-F birds had the highest levels of CD3^+^CD8α^+^ cells among all lines at baseline, followed by Line-8, which had 58.4 and 42.0% more cells than Ghs and Sp-21.1 lines, respectively (65.0% Ghs, 16.0% Line-8, and 51.3% Sp-21.1, respectively; *p* < 0.0001; Figure 1E). In turn, Sp-21.1 birds had 28.3% more CD3^+^CD8α^+^ cells than the Ghs line (*p* < 0.0001; Figure 1E).

At 6 hpi, the genetic line and injection interaction was significant for each analyzed PBMC immune cell population (*p* ≤ 0.02, Figures 1A–E). Within LPS-stimulated birds, Sp-21.1 and AIL-F had more circulating monocyte/macrophage^+^ cells by 45.7 and 48.7%, respectively, than their control counterparts at 6 hpi (*p* < 0.00001, Figure 1A). However, Ghs LPS-stimulated birds had 59.2% fewer monocyte/macrophage^+^ cells than their control birds and Line-8 birds remained similar at 6 hpi (*p* < 0.0001, Figure 1A). LPS significantly decreased circulating CD3^+^ cells compared to control across all genetic lines (34.0% Ghs, 68.1% Line-8, 48.1% Sp-21.1, and 55.4% AIL-F; *p* < 0.0001, Figure 1B). Within CD3^+^ subpopulations at 6 hpi, Ghs, Line-8, and Sp-21.1 LPS-stimulated birds had more CD3^+^CD1.1^+^ cells compared to respective control, while AIL-F birds remained similar (37.0, 42.7, and 25.4%, respectively; *p* < 0.0001, Figure 1C). LPS significantly decreased CD3^+^CD4^+^ cells in the AIL-F line by 35.3%, while all genetic lines remained similar across treatments (*p* = 0.02, Figure 1D). Line-8 LPS-stimulated birds had 34.5% fewer CD3^+^CD8α^+^ cells than control, while LPS increased CD3^+^CD8α^+^ cells in Sp-21.1 LPS-stimulated birds by 61.5%, and Ghs and AIL-F birds remained similar (*p* < 0.0001, Figure 1E).

At 24 hpi, genetic line and injection interaction remained significant for all PBMC immune cell populations analyzed except CD3^+^ and CD3^+^CD4^+^ cells (Figures 1A–E). LPS-stimulated Ghs birds had 57.9% more monocyte/macrophage^+^ cells, while Line-8 and Sp-21.1 birds remained similar at 24 hpi (*p* = 0.02, Figure 1A). Monocyte/macrophage^+^ cells also remained elevated from 6 hpi in AIL-F, with increased monocyte/macrophage^+^ cells in AIL-F LPS birds by 46.4% compared to their respective control birds at 24 hpi (*p* = 0.02). In CD3 + subpopulations at 24 hpi, AIL-F LPS-stimulated birds had 17.2% fewer CD1.1^+^ cells and Line-8 LPS-stimulated birds had 34.5% fewer CD3^+^CD8α^+^ cells compared to their control counterparts, while LPS challenge did not impact Ghs and Sp-21.1 lines (*p* = 0.02 and *p* = 0.01, respectively; Figures 1C,E). While genetic line and injection interaction did not affect CD3^+^ and CD3^+^CD4^+^ cells at 24 hpi, the genetic line main effect was significant but not injection (*p* < 0.0001, Supplementary Table 1).

Within the main effects of genetic line, Line-8 exhibited the highest levels of CD3^+^ cells among all lines (55.1% Ghs, 38.2% Sp-21.1, and 21.7% AIL-F) at 24 hpi, followed by AIL-F, which had 42.6 and 21.0% more cells than Ghs and Sp-21.1, respectively (*p* < 0.0001). In turn, Sp-21.1 had 27.3% more cells than Ghs at 24 hpi (*p* < 0.0001). Within CD3^+^ subpopulations, Sp-21.1 had more CD3^+^CD4^+^ cells than Ghs, Line-8, and AIL-F lines (26.9, 14.3, and 12.2%, respectively; *p* < 0.0001). In addition, Line-8 and AIL-F birds 14.6 and 16.7% more CD3^+^CD4^+^ cells than Ghs birds (*p* < 0.0001).

### 3.3 Immunometabolic phenotype

**Figure 2:**
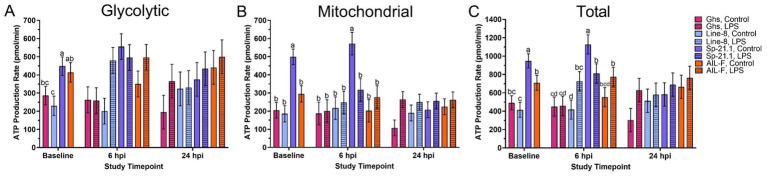
**(A)** Glycolytic, **(B)** mitochondrial, and **(C)** total ATP production of peripheral blood mononuclear isolated from 4 genetic lines ± 1 mg/kg intramuscular LPS injection at baseline, 6 hpi, and 24 hpi. Data represent the mean ± SEM (*n* = 10 birds/genetic line at baseline and *n* = 5/treatment of each genetic line at 6 hpi and 24 hpi). Note that the y-axis is larger in panel **(C)**. Different letters denote significant differences within a timepoint (*p* ≤ 0.05).

Before LPS injection, Sp-21.1 birds produced more glycolytic ATP than Ghs and Line-8 birds (36.1 and 48.7%, respectively; *p* = 0.02; Figure 2A, Supplementary Table 2). Sp-21.1 birds also produced 41.0–62.9% more mitochondrial ATP and 25.2–56.2% more total ATP than all other lines (*p* < 0.0001 and *p* = 0.0001, respectively; Figures 1B,C). In the AIL-F line, birds had produced 44.5% more glycolytic ATP, resulting in a greater total ATP production of 41.4%, than Line-8 birds (*p* = 0.02 and *p* = 0.0001, respectively; Figures 2A,C).

At 6 hpi, genetic line and injection interaction impacted mitochondrial and total ATP production but not glycolytic (Figure 2, *p* ≤ 0.05). In the Sp-21.1 line, LPS-stimulated birds produced 44.7% less mitochondrial ATP and 28.0% less total ATP than their control counterparts (*p* = 0.05 and *p* = 0.03, respectively; Figures 2B,C). However, total ATP production increased by 42.7% in Line-8 LPS-stimulated birds compared to Line-8 control birds (*p* = 0.03, Figure 2C). The main effect of genetic line significantly impacted ATP production at 6 hpi, where Sp-21.1 birds had produced more ATP via glycolysis than Ghs and Line-8 birds (50.4 and 35.3%, respectively; *p* = 0.005, Figure 1A). In addition, Sp-21.1 birds produced 46.1–56.4% more mitochondrial and 31.6–53.1% more total ATP than all genetic lines at 6 hpi (*p* = 0.001 and *p* = 0.0002, respectively; Figures 2B,C). At 24 hpi, the significant main effect of injection resulted in LPS-stimulated birds producing 29.4% greater mitochondrial ATP than control birds (*p* = 0.02, Figure 2B). No other effects were significant at 24 hpi within ATP production (*p* ≥ 0.51 and *p* ≥ 0.22, respectively; Figure 2, Supplementary Table 2).

When comparing basal measures of the glycolytic rate assay prior to LPS injection, Sp-21.1 birds had greater basal PER compared to all other genetic lines (50.1% Ghs, 60.1% Line-8, and 42.1% AIL-F; *p* = 0.02, Table 3). Sp-21.1 also had a greater basal glycolytic rate compared to Ghs and Line-8 birds, with AIL-F birds falling intermediate (49.8 and 62.0%, respectively; *p* = 0.02). Following Rot/AA injection at baseline, compensatory glycolysis was 286.7–380.6 pmol/min greater in Sp-21.1 birds than all other lines (*p* = 0.02). Following 2-DG injection, post-2-DG acidification was greatest in Sp-21.1, while all other lines remained similar (66.3–75.4%, *p* = 0.02). However, at 6 hpi, no significant differences were observed in basal PER, basal glycolysis, compensatory glycolysis, and post 2-DG acidification due to genetic line, LPS injection, or their interaction (*p* ≥ 0.05, Table 3). By 24 hpi, genetic line and LPS injection had a significant effect on basal measurements, while interaction between genetic line and injection did not. AIL-F birds had greater basal PER than Sp-21.1 and Ghs at 24 hpi, with Line-8 birds falling intermediate (37.0 and 41.6%, respectively; *p* = 0.04, Table 3). AIL-F birds also had greater basal PER than all other genetic lines (38.3% Ghs, 34.4% Line-8, and 43.4% Sp-21.1; *p* = 0.04). LPS injection increased basal PER by 25.8% compared to control birds at 24 hpi (*p* = 0.05, Table 3). Following drug injections, compensatory glycolysis and post-2-DG acidification remained similar across genetic lines and treatments at 24 hpi (*p* ≥ 0.07).

**Table 3:** Basal proton efflux rate (PER), basal glycolysis, compensatory glycolysis, and post 2-DG acidification of all genetic lines ± 1 mg/kg intramuscular LPS injection at baseline, 6 hpi, and 24 hpi.

Measure (pmol/min)	Ghs		Line-8		Sp-21.1		AIL-F				Adj. *p*-value	
	Control	LPS	Control	LPS	Control	LPS	Control	LPS	Pooled SEM	Line^1^	Trt^2^	Line^1^ x Trt^2^
Basal PER^3^												
Baseline	237.76^b^	-	190.16^b^	-	476.87^a^	-	275.87^b^	-	64.38	0.02	-	-
6 hpi	201.67	188.47	217.02	388.83	357.02	310.16	230.13	362.56	64.51	0.17	0.19	0.27
24 hpi	115.23	273.37	158.62	298.70	181.15	238.18	367.46	298.26	49.97	0.04	0.05	0.12
Basal glycolysis												
Baseline	217.22^b^	-	164.43^b^	-	432.40^a^	-	242.11^ab^	-	61.55	0.02	-	-
6 hpi	178.39	162.87	182.00	359.90	297.22	273.19	206.13	330.29	59.19	0.21	0.13	0.25
24 hpi	104.09	240.79	135.13	264.83	162.86	213.37	338.76	270.64	47.65	0.04	0.08	0.15
Compensatory glycolysis												
Baseline	305.17^b^	-	267.39^b^	-	648.02^a^	-	361.28^b^	-	88.68	0.02	-	-
6 hpi	247.97	212.62	303.36	445.22	477.77	382.31	265.61	421.50	80.90	0.11	0.47	0.32
24 hpi	139.37	310.28	193.95	361.21	220.68	271.86	404.96	328.25	59.27	0.11	0.07	0.15
Post 2-DG												
Baseline	158.99^b^	-	115.88^b^	-	471.25^a^	-	157.51^b^	-	83.80	0.02	-	-
6 hpi	93.20	40.15	89.50	82.00	152.58	73.25	78.35	73.80	25.29	0.30	0.052	0.39
24 hpi	54.05	82.66	67.60	105.96	62.10	81.89	148.86	83.95	24.35	0.21	0.75	0.17

Data represent the mean ± SEM (*n* = 10 birds/genetic line at baseline and *n* = 5/treatment of each genetic line at 6 hpi and 24 hpi). Different letter superscripts within a timepoint are significantly different (*p* ≤ 0.05).

1 Line = Genetic line main effect.

2 Trt = Injection main effect.

3 PER = Proton Efflux Rate.

### 3.4 Plasma C-reactive protein

At 6hpi, LPS-stimulated birds had significantly increased CRP concentrations by 54.0% compared to control birds (*p* ≥ 0.02), a difference which had resolved by 24 hpi. No other differences were found due to genetic line or the interaction at any timepoint (*p* ≥ 0.28, Table 4).

**Table 4:** C-reactive protein (CRP) expression of all genetic line birds ± 1 mg/kg intramuscular LPS injection at baseline, 6 hpi, and 24 hpi.

CRP (ng/mL)	Ghs		Line-8		Sp-21.1		AIL-F			Adj. *p*-value		
	Control	LPS	Control	LPS	Control	LPS	Control	LPS	Pooled SEM	Line^1^	Trt^2^	Line^1^ x Trt^2^
Baseline	3,070.5	-	4,930.0	-	4,264.2	-	3,605.3	-	769.6	0.39	-	-
6 hpi	5,408.9	17,178.0	6,106.7	15,044.0	5,413.3	9,115.6	4,562.7	5,389.3	3,606.3	0.28	0.02	0.45
24 hpi	15,769.0	8,900.0	7,271.1	7,680.0	1,431.7	7,184.4	3,071.7	15,461.0	5,165.3	0.51	0.43	0.30

Data represent the mean ± SEM (*n* = 10 birds/genetic line at baseline and *n* = 5/treatment of each genetic line at 6 hpi and 24 hpi). Different letter superscripts within a timepoint are significantly different (*p* ≤ 0.05).

1 Line = Genetic line main effect.

2 Trt = Injection main effect.

## 4 Discussion

Within the LPS model used in this study, differences in several experimental variables were found due to genetic line and LPS administration. When designing this experiment, different LPS injection strategies were considered. In alignment with prior work and due to some of the unknowns with response to LPS, a 1 mg/kg dose was selected. At 6 hpi, LPS-induced trends in cloacal temperature aligned with findings from previous work in our lab, where 1 mg/kg BW intramuscular LPS injection trended to decrease body temperature by 0.20°C compared to the 0.27°C decrease observed in the current study (30). In addition to the physiological stress-induced response due to LPS injection, increased CRP concentrations observed in LPS-stimulated birds confirmed that the model used in this study was appropriate. However, an alternative administration route or increase in dosage will be considered in future studies to capture LPS-induced changes in body temperature.

In other LPS models, de Boever et al. and Jones et al. used a similar 1 mg/kg LPS dosage (*E. coli* O127: B8) but administered it intravenously rather than intramuscularly (32, 33). De Boever et al. saw a fever response develop by 5 hpi in 3-wk and 5-wk-old Ross broilers, whereas Jones et al. saw a significant increase in body temperature as early as 2 h post-LPS injection in 5-wk-old broilers (32, 33). Genotype has also been shown to influence body temperature as early as 3 h following a 2 mg/kg intramuscular LPS injection [*E. coli* O111: B4, (13)].

Prior to LPS administration, significant differences in immune cell populations were observed, indicating a strong genetic influence on baseline immune profiles and also likely a part of the genetic line-specific differences in response to both bacterial and viral pathogens (16–24, 31). Although Ghs and Line-8 birds exhibited higher monocyte/macrophage^+^ cell populations at baseline, the percentage differences in the absence of a challenge may not necessarily equate to biological relevance. Line-8 birds also had more CD3^+^ cells at baseline compared to all other lines, which may indicate that these birds have a relatively larger T-cell pool to activate within their total immune cell population. Line-8 did not display remarkable differences at baseline in the Seahorse assay, while Sp-21.1 birds displayed significantly increased baseline mitochondrial ATP production than all other lines (Figure 2B). Baseline plasma CRP concentrations confirmed that inflammation levels were similar across genetic lines despite genetic-related differences in immune cell profile and immunometabolism. Therefore, it is important to apply a whole-animal challenge to begin to query immune function.

PBMC from all genetic lines demonstrated the ability to support immune function by effectively switching to a more glycolytic state when forced to by Rot/AA injection at baseline. Sp-21.1 was the most glycolytic from both anaerobic and mitochondrial production standpoints, while the remaining lines did not differ in preference for aerobic or anaerobic. Significant differences were observed among all 4 genetic lines in PER or glycoPER at baseline. For example, Sp-21.1 had the greatest PER, compensatory glycolysis, and post-2-DG acidification compared to all other lines. This result may indicate that Sp-21.1 PMBC may have been more metabolically active compared to all other lines at baseline. General metabolic profiles and cellular responses to assay inhibitors suggested that the birds could produce ATP independent of mitochondrial respiration at baseline unstressed states in the presence of a mitochondrial respiration inhibitor. Comparable results by Meyer et al. showed significantly increased mitochondrial respiration observed in ~32-wk-old Line-8 hens when compared to Ghs-6 and advanced broiler intercross (broiler x Fayoumi M-15.2) lines but not in comparison to ~32-wk-old Sp-21.1 hens (27). Therefore, further research is needed to investigate the underlying mechanisms driving the enhanced oxidative phenotype observed in Sp-21.1. This research may provide insights into potential genetic factors contributing to improved oxidative capacity, overall immune health, and disease resistance in this line.

At 6 hpi, the interaction between genetic line and LPS injection significantly influenced all PBMC immune cell populations, further highlighting the diverse immune responses among the different lines. CD3^+^ circulating T cells were significantly reduced in all lines, while LPS increased the CD3^+^CD1.1^+^ subset in all lines except AIL-F at 6 hpi (Figure 1). This increase in CD1.1^+^ cells was anticipated as Elmore et al. observed a similar outcome at 24 h post-1 mg/kg LPS injection, where aged (~133-week-old) White Leghorn roosters administered LPS had a higher number of CD1.1^+^ cells compared to their control counterparts (30). Within CD3^+^ subpopulations at 6 hpi, Sp-21.1 birds had increased CD1.1^+^ and CD3^+^CD8α^+^, suggesting better antigen presentation, cytokine signaling, and immune modulation than all other lines (34–36). Meanwhile, AIL-F birds had more CD3^+^CD8α^+^ cells compared to all other lines, which could correlate with enhanced cytotoxic responses and adaptive immunity (35, 36). While research directly comparing immune cell profiles of these legacy inbred lines is limited, Fries-Craft et al. showed that 21-d-old Fayoumi M-5.1 chicks had significantly more monocyte/macrophage^+^, CD3^+^, and CD3^+^CD8α^+^ cells at baseline than Ghs Leghorn lines (31). Therefore, similar baseline immune profiles to the M-5.1 line were expected in the advanced commercial broiler and Fayoumi intercross in the current study but were likely not observed due to known age-related immune decline (37).

LPS injection increased monocyte/macrophage^+^ cells in Sp-21.1 and AIL-F birds, indicating a strong innate immune response in these lines at 6 hpi (38). In contrast, Ghs birds had a significant reduction in monocyte/macrophage^+^ cells at 6 hpi, which may suggest a redistribution of T cells from the bloodstream to sites of inflammation, such as the LPS injection sites in the breast and thigh muscles, highlighting line-specific variations in immune regulation. A limitation of this work is that the response at localized injection sites was not measured, and this could represent an avenue for future research based on the injection model and immune cell movement to tissues. Metabolically, ATP production varied among genetic lines at 6 hpi, with sustained elevated mitochondrial and total ATP production from baseline measurements. However, genetic line, LPS status, or their interaction at 6 hpi did not significantly impact glycolytic activity. This suggests that genetic line effects on PER, basal and compensatory glycolysis, and post-2-DG acidification were resolved from baseline.

By 24 hpi, cloacal temperature and circulating CRP concentrations were resolved, suggesting possible LPS clearance. This aligns with previous findings, where body temperatures in 12-wk-old cockerels normalized as early as 14 h post 2 mg/kg intramuscular LPS injection (13). However, despite apparent recovery, distinct immune and metabolic profiles were observed across genetic lines and treatments, providing further insight into cell management and recovery 24 h post immune challenge. For example, Sp-21.1 and Line-8 birds had relatively stable monocyte/macrophage^+^ populations and lower basal glycolytic activity at 24 hpi. This may reflect a more efficient LPS resolution and indicate an ability to effectively clear the stimulus without sustaining unnecessary immune activation or incurring additional metabolic costs.

In contrast, AIL-F birds sustained elevations in monocyte/macrophage^+^ populations from 6 hpi and exhibited the highest basal PER within glycolytic activity at 24 hpi. These outcomes suggest that AIL-F PBMC may have been in a prolonged state of immune activation and heightened energy demand, which may indicate a continued inflammatory response or delayed resolution (39, 40). While feed intake and other performance measures were not evaluated during the current study, the sustained immune and metabolic impact following LPS injection may lead to greater performance losses in AIL-F birds due to prolonged resource allocation toward immune processes (8, 9). In Ghs birds, a delayed increase in monocyte/macrophage^+^ populations was observed at 24 hpi due to LPS injection, while their metabolic activity had relatively normalized by 24 hpi. This suggests that Ghs PBMC were able to resolve LPS 24 hpi despite delayed immune cell recruitment in comparison to other lines. While previous work from this research group has investigated PBMC immune and metabolic response following *Eimeria* challenge in Fayoumi and Ghs lines (M-5.1, Ghs-6, and Ghs-13; (31)), future studies should investigate the impact of repeated immune challenges on immune profile and immunometabolic recovery.

A drawback of the current experiment was that it was not repeated with additional cohorts due to the limited availability of these specific genetic lines. Previous work has been conducted in these lines with similar numbers across different ages (30, 31). Therefore, assay repetitions were a key consideration in the study design, and the number of assays per bird was intentionally increased to enhance the robustness and reliability of the data. This approach allowed for a thorough assessment of both metabolic and immune responses across the genetic lines and this method has been used successfully in the past. Several peer-reviewed studies have employed similar group sizes, supporting the general acceptance of using approximately 10 birds per group for this type of research. For example, 10–15 birds per group has been used by others previously for flow cytometry (41), while 6 birds/treatment were used for serum assays (10). While repeating the trials in succession could be ideal, the assays and statistical analyses applied in this study provide sound and reproducible insights into the immunometabolic responses across genetic backgrounds.

Overall, genetic line played a significant role in immune cell populations and immunometabolic responses to systemic LPS challenge. The Sp-21.1 line displayed a strong T cell response and glycolytic metabolic profile that could be advantageous when supporting rapid, high-energy-demand immune responses. In contrast, the AIL-F line had delayed shifts in immune cell populations and metabolism. Line-8 had increased ATP production, reflecting distinct immune-metabolic trade-offs. Despite differences among genetic lines, metabolic recovery across all genetic lines by 24 hpi suggests that the cost of energy by LPS was transient. These findings suggest that genetic background influences the scale and efficiency of immune activation and resolution. Future work incorporating increased LPS doses, varying bird ages, alternative LPS administration routes, and expanded sampling timepoints are warranted further to investigate the effects of LPS injection on cellular metabolism. Examining the long-term effects of metabolic phenotypes on production traits may offer valuable information for breeding strategies to optimize health and production efficiency in commercial poultry systems.

## Data Availability

The raw data supporting the conclusions of this article will be made available by the authors, without undue reservation.
