# Supplementary material for: Immune cell profile and metabolic preference following intramuscular lipopolysaccharide injection of highly inbred and advanced intercross genetic lines
Source: Front Vet Sci. 2025 Jun 3;12:1592021. doi: 10.3389/fvets.2025.1592021 (PMC12170644; doi:10.3389/fvets.2025.1592021)
Supplement: Supplementary file 1 [file Table_1.docx]

Supplementary Material

**Supplemental Table 1.** Monocyte/Macrophage^+^, total CD3^+^, CD3^+^ CD1.1^+^, CD3^+^ CD4^+^, and CD3^+^ CD8α cells isolated from peripheral blood mononuclear cells of genetic line (Ghs, Line-8, Sp-21.1, and AIL-F) ± 1 mg/kg intramuscular LPS injection at baseline, 6 hpi, and 24 hpi.

|  | Ghs | | Line-8 | | | Sp-21.1 | | AIL-F | | |  | Adj. *P*-value | | |
| --- | --- | --- | --- | --- | --- | --- | --- | --- | --- | --- | --- | --- | --- | --- |
| Measure (%) | Control | LPS | | Control | LPS | Control | LPS | | Control | LPS | SEM | Line^1^ | Trt^2^ | Line^1^ x Trt^2^ |
| Monocyte/  macrophage |  |  | |  |  |  |  | |  |  |  |  |  |  |
| Baseline | 6.57^a^ | - | | 6.99^a^ | - | 5.11^b^ | - | | 5.29^b^ | - | 0.40 | 0.002 | - | - |
| 6 hpi | 6.12^bc^ | 2.50^d^ | | 6.07^bc^ | 5.01^c^ | 7.66^b^ | 14.11^a^ | | 6.95^bc^ | 13.56^a^ | 0.85 | <0.0001 | 0.0003 | <0.0001 |
| 24 hpi | 5.93^cde^ | 14.08^a^ | | 10.03^b^ | 9.70^bc^ | 5.60^de^ | 8.95^bcd^ | | 5.12^e^ | 9.56^bc^ | 1.37 | 0.06 | <0.0001 | 0.02 |
| CD3^+^ |  |  | |  |  |  |  | |  |  |  |  |  |  |
| Baseline | 16.14^d^ | - | | 32.86^a^ | - | 26.46^b^ | - | | 22.71^c^ | - | 1.03 | <0.0001 | - | - |
| 6 hpi | 21.45^c^ | 14.15^d^ | | 42.59^a^ | 13.60^d^ | 26.44^c^ | 13.73^d^ | | 34.73^b^ | 15.48^d^ | 2.06 | <0.0001 | <0.0001 | <0.0001 |
| 24 hpi | 19.60 | 17.21 | | 37.75 | 44.17 | 22.52 | 28.11 | | 31.68 | 32.43 | 2.50 | <0.0001 | 0.14 | 0.25 |
| CD1.1^+^ |  |  | |  |  |  |  | |  |  |  |  |  |  |
| Baseline | 18.34^b^ | - | | 21.02^b^ | - | 26.33^a^ | - | | 20.49^b^ | - | 1.01 | <0.0001 | - | - |
| 6 hpi | 17.52^d^ | 27.81^bc^ | | 22.96^cd^ | 40.06^a^ | 31.27^b^ | 41.94^a^ | | 28.03^bc^ | 24.88^c^ | 2.13 | <0.0001 | <0.0001 | <0.0001 |
| 24 hpi | 22.32^c^ | 23.60^c^ | | 21.23^c^ | 24.14^c^ | 37.96^a^ | 36.23^a^ | | 30.01^b^ | 24.85^c^ | 1.56 | <0.0001 | 0.49 | 0.02 |
| CD4^+^ |  |  | |  |  |  |  | |  |  |  |  |  |  |
| Baseline | 35.50^ab^ | - | | 33.96^bc^ | - | 38.51^a^ | - | | 31.16^c^ | - | 1.26 | 0.001 | - | - |
| 6 hpi | 34.80^bc^ | 32.68^cd^ | | 37.70^abc^ | 31.83^cd^ | 42.52^a^ | 39.12^ab^ | | 41.27^a^ | 26.70^d^ | 2.16 | 0.003 | <0.0001 | 0.02 |
| 24 hpi | 37.33 | 31.68 | | 37.98 | 37.04 | 42.00 | 45.57 | | 39.58 | 37.33 | 1.98 | <0.0001 | 0.96 | 0.49 |
| CD8α^+^ |  |  | |  |  |  |  | |  |  |  |  |  |  |
| Baseline | 8.86^d^ | - | | 21.30^b^ | - | 12.35^c^ | - | | 25.35^a^ | - | 0.89 | <0.0001 | - | - |
| 6 hpi | 7.04^d^ | 11.21^cd^ | | 18.65^b^ | 12.21^c^ | 9.77^cd^ | 25.37^a^ | | 25.42^a^ | 28.39^a^ | 1.62 | <0.0001 | 0.001 | <0.0001 |
| 24 hpi | 6.39^c^ | 5.95^c^ | | 20.24^a^ | 14.54^b^ | 12.76^b^ | 12.87^b^ | | 21.62^a^ | 20.85^a^ | 1.20 | <0.0001 | 0.03 | 0.01 |

Data represents the mean ± SEM (n=10 birds/genetic line at baseline and n=5/treatment of each genetic line at 6 hpi and 24 hpi). Different letter superscripts within a timepoint are significantly different (*p* ≤ 0.05).

^1^Line = Genetic line main effect

^2^Trt = Injection main effect

**Supplemental Table 2.** Glycolytic, mitochondrial, and total ATP production by genetic lines ± 1 mg/kg intramuscular LPS injection at baseline, 6 hpi, and 24 hpi.

|  | Ghs | | Line-8 | | Sp-21.1 | | AIL-F | |  | Adj. *P*-value | | |
| --- | --- | --- | --- | --- | --- | --- | --- | --- | --- | --- | --- | --- |
| ATP Production (pmol/min) | CON | LPS | CON | LPS | CON | LPS | CON | LPS | Pooled SEM | Line^1^ | Trt^2^ | Line^1^ xTrt^2^ |
| Glycolytic |  |  |  |  |  |  |  |  |  |  |  |  |
| Baseline | 286.4^bc^ | - | 230.0^c^ | - | 448.2^a^ | - | 414.2^ab^ | - | 52.34 | 0.02 | - | - |
| 6 hpi | 263.0 | 258.4 | 200.4 | 479.7 | 555.9 | 495.4 | 350.5 | 496.5 | 71.00 | 0.005 | 0.08 | 0.09 |
| 24 hpi | 195.3 | 365.3 | 323.4 | 330.7 | 375.4 | 434.1 | 441.1 | 500.3 | 92.75 | 0.22 | 0.28 | 0.85 |
| Mitochondrial |  |  |  |  |  |  |  |  |  |  |  |  |
| Baseline | 204.8^b^ | - | 185.3^b^ | - | 499.4^a^ | - | 294.7^b^ | - | 43.95 | <0.0001 | - | - |
| 6 hpi | 187.0^b^ | 200.6^b^ | 216.5^b^ | 247.3^b^ | 572.2^a^ | 316.6^b^ | 202.1^b^ | 277.2^b^ | 62.13 | 0.001 | 0.44 | 0.05 |
| 24 hpi | 107.4 | 264.7 | 189.8 | 249.6 | 207.7 | 256.1 | 224.4 | 262.4 | 43.45 | 0.60 | 0.02 | 0.51 |
| Total |  |  |  |  |  |  |  |  |  |  |  |  |
| Baseline | 491.2^bc^ | - | 415.3^c^ | - | 947.6^a^ | - | 709.0^b^ | - | 79.29 | <0.0001 | - | - |
| 6 hpi | 450.0^cd^ | 459.0^cd^ | 416.9^d^ | 727.0^bc^ | 1128.0^a^ | 811.9^b^ | 552.6^bcd^ | 773.7^b^ | 104.80 | 0.002 | 0.46 | 0.03 |
| 24 hpi | 302.8 | 630.0 | 513.1 | 580.3 | 583.0 | 690.2 | 665.4 | 762.7 | 127.23 | 0.27 | 0.12 | 0.73 |

Data represents the mean ± SEM (n=10 birds/genetic line at baseline and n=5/treatment of each genetic line at 6 hpi and 24 hpi). Different letter superscripts within a timepoint are significantly different (*p* ≤ 0.05).

^1^Line = Genetic line main effect

^2^Trt = Injection main effect
